# Why Monoamine Oxidase B Preferably Metabolizes *N*-Methylhistamine over Histamine: Evidence from the Multiscale Simulation of the Rate-Limiting Step

**DOI:** 10.3390/ijms23031910

**Published:** 2022-02-08

**Authors:** Aleksandra Maršavelski, Janez Mavri, Robert Vianello, Jernej Stare

**Affiliations:** 1 Computational Organic Chemistry and Biochemistry Group, Ruđer Bošković Institute, Bijenička 54, HR-10000 Zagreb, Croatia; aleksandra.marsavelski@chem.pmf.hr; 2 Department of Chemistry, Faculty of Science, University of Zagreb, Horvatovac 102a, HR-10000 Zagreb, Croatia; 3 Theory Department, National Institute of Chemistry, Hajdrihova 19, SI-1000 Ljubljana, Slovenia; janez.mavri@ki.si; 4 Laboratory for the Computational Design and Synthesis of Functional Materials, Ruđer Bošković Institute, Bijenička 54, HR-10000 Zagreb, Croatia

**Keywords:** histamine, *N*-methylhistamine, selectivity, metabolic pathway, monoamine oxidase B, rate constant, activation free energy, multiscale molecular simulations, QM/MM, empirical valence bond, DFT calculations

## Abstract

Histamine levels in the human brain are controlled by rather peculiar metabolic pathways. In the first step, histamine is enzymatically methylated at its imidazole *N*^τ^ atom, and the produced *N*-methylhistamine undergoes an oxidative deamination catalyzed by monoamine oxidase B (MAO-B), as is common with other monoaminergic neurotransmitters and neuromodulators of the central nervous system. The fact that histamine requires such a conversion prior to oxidative deamination is intriguing since MAO-B is known to be relatively promiscuous towards monoaminergic substrates; its in-vitro oxidation of *N*-methylhistamine is about 10 times faster than that for histamine, yet this rather subtle difference appears to be governing the decomposition pathway. This work clarifies the MAO-B selectivity toward histamine and *N*-methylhistamine by multiscale simulations of the rate-limiting hydride abstraction step for both compounds in the gas phase, in aqueous solution, and in the enzyme, using the established empirical valence bond methodology, assisted by gas-phase density functional theory (DFT) calculations. The computed barriers are in very good agreement with experimental kinetic data, especially for relative trends among systems, thereby reproducing the observed MAO-B selectivity. Simulations clearly demonstrate that solvation effects govern the reactivity, both in aqueous solution as well as in the enzyme although with an opposing effect on the free energy barrier. In the aqueous solution, the transition-state structure involving histamine is better solvated than its methylated analog, leading to a lower barrier for histamine oxidation. In the enzyme, the higher hydrophobicity of *N*-methylhistamine results in a decreased number of water molecules at the active side, leading to decreased dielectric shielding of the preorganized catalytic electrostatic environment provided by the enzyme. This renders the catalytic environment more efficient for *N*-methylhistamine, giving rise to a lower barrier relative to histamine. In addition, the transition state involving *N*-methylhistamine appears to be stabilized by the surrounding nonpolar residues to a larger extent than with unsubstituted histamine, contributing to a lower barrier with the former.

## 1. Introduction

Histamine is a biologically important amine that exerts its effect through interaction with histamine receptors (H_1_R, H_2_R, H_3_R, and H_4_R) [1,2,3]. Histamine is a mediator of many different physiological processes, such as the contraction of smooth muscle tissues, dilatation of blood vessels, and gastric acid secretion. It also plays important roles in neurotransmission and immunomodulation. Because of its essential roles in the human body, regulation of histamine level is of paramount importance [4,5]. There are two pathways of histamine degradation. In the peripheral organs, diamine oxidase (DAO) catalyzes a direct oxidative deamination of histamine to imidazole acetaldehyde [6,7]. Under normal circumstances, imidazole acetaldehyde is quickly oxidized to imidazole acetic acid [8]. In the other pathway prevalent in the brain, histamine *N*-methyltransferase (HNMT) catalyzes the transfer of a methyl group to the secondary imidazole amine forming *N*-methylhistamine, rendering it inactive at histamine receptor sites [9]. This compound is then metabolized by monoamine oxidase B (MAO-B) forming *N*-methylimidazole acetaldehyde [10]. MAO-B is a promiscuous enzyme that acts on many different biogenic amines, such as dopamine, serotonin, phenylethylamine, benzylamine, epinephrine, and norepinephrine [11,12]. On the other hand, MAO-B distinguishes between two similar compounds, histamine and its methylated form, *N*-methylhistamine, showing both in vivo and in vitro selective activity towards the latter compound [13,14,15]. The fact that MAO-B prefers *N*-methylhistamine over histamine pinpoints its considerable selectivity towards two compounds differing only in a single methyl group distant from the reactive ethylamino center. 

Under normal conditions, histamine is not a substrate of MAO-B. However, research of histamine metabolism in rat brain pretreated with metoprine, an inhibitor of HNMT that blocks the methylation pathway, reveals the presence of (unmethylated) imidazole acetic acid, a metabolite characteristic of direct oxidation of histamine [16]. Since DAO is not present in the brains of mammals [17], the study implies alternative histamine metabolism when the HNMT enzyme is inhibited. Interestingly, Edmondson and co-workers performed a series of in vitro experiments and, after experiencing many difficulties, reported only an approximate value of *K*_m_ ≈ 4000 μM for histamine and MAO-B, while the value for *N*-methylhistamine was precisely measured at *K*_m_ = 166.0 ± 8.1 μM [13].

The mechanism of an enzymatic reaction and its specificity is usually elucidated from the differences in mechanistic aspects for each possible substrate, which provide unambiguous thermodynamic and kinetic insight into the catalytic process. Unfortunately, mechanistic studies are often not experimentally viable. Therefore, to reveal determinants behind both the activity and substrate selectivity of this, otherwise promiscuous, enzyme, we have simulated an enzymatically catalyzed reaction of oxidative deamination of both substrates, histamine (HIS) and *N*-methylhistamine (NMH). 

The selectivity of MAO enzymes is a crucial feature for their therapeutic application [18]. When it comes to inhibitors, it turns out that MAOs evade promiscuity and become very selective. Inhibitors that act on MAO-A are used to treat depression, due to their ability to raise serotonin concentrations, whereas MAO-B inhibitors decrease dopamine degradation and improve motor control in patients with Parkinson disease. Despite this functional importance, the factors affecting MAO selectivity are still poorly understood. In our previous work, we have addressed this issue by using a combination of classical molecular dynamics simulations, evaluations of binding free energy using the molecular mechanics Poisson–Boltzmann surface area (MM–PBSA) methodology, and quantum mechanical calculations within a cluster model of an enzyme [19]. We showed that dominant selectivity contributions are offered by more favorable active-site binding and reaction exergonicity for NMH together with a lower activation free energy for the enzymatic reaction, evaluated within a truncated model of the enzyme. However, for a full account of factors contributing to the MAO-B selectivity in this very curious and challenging case of two very similar molecules, a proper treatment of the chemical reactivity of these substrates is required. Therefore, the purpose of this paper is to advance the previous study and consider full enzyme dimensionality with thermal averaging in the study of the chemical reactivity of MAO-B towards HIS and NMH and to inspect how subtle differences in their interactions with the enzyme active site govern the selectivity. 

Probably the most effective current way to simulate enzymatic reactions is provided by the empirical valence bond (EVB) approach of Warshel and coworkers [20,21,22,23]. Technically, EVB is a multiscale quantum/classical (QM/MM) methodology, yet with a special description of the quantum part. Namely, in contrast to the most popular QM/MM approaches, rather than quantizing electronic structure, EVB imposes quantum principles on empirical valence states, typically representing reactants and products of a chemical reaction. Because of simplicity (typically using only two distinguishable quantum states), the approach is fast and allows for simulation timescales typical of classical methods, facilitating efficient thermal averaging and giving rise to well-converged reaction-free energy profiles. However, prior to application to a system of interest (e.g., reaction in an enzyme active site), the tunable EVB parameters need to be calibrated against a reference reaction for which the kinetic and thermodynamic quantities are known in advance. EVB proved to be a robust and reliable tool to simulate chemical reactions catalyzed by enzymes [21,23,24,25,26,27]. It has been successful in explaining the catalytic mechanism of many different enzymes, and it has contributed significantly to the improved understanding of their functionality, including their selectivity [28,29,30]. Importantly, the EVB approach has been designed in conjunction with the postulate that the catalytic role of enzymes derives from preorganized electrostatics [25,31]. This view has been recently supported by the analysis of the topology of the electron charge density [32] and of the electric fields within the enzyme active site [33,34], as well as by embedding the reacting moiety treated by quantum chemistry protocols into the enzymatic environment represented by point charges [35]. 

**Scheme 1:**
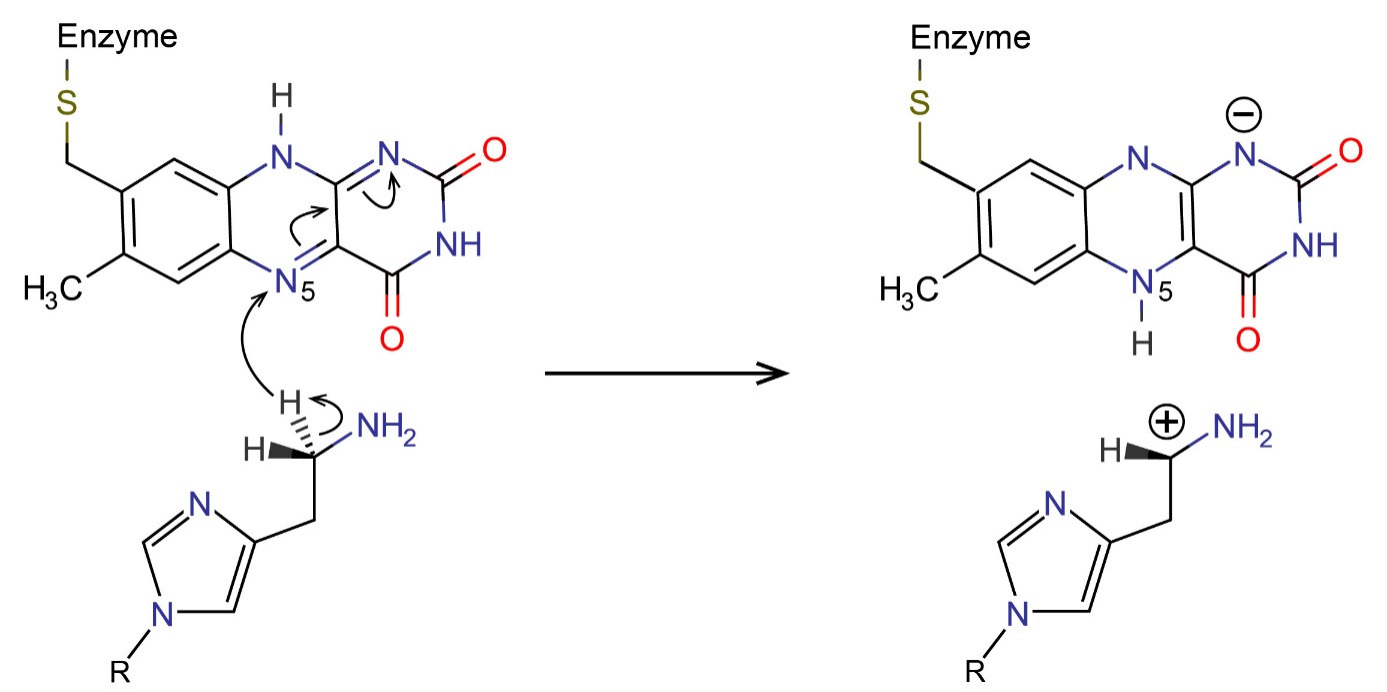
Rate limiting step of histamine (R = H) and *N*-methylhistamine (R = CH_3_) oxidation catalyzed by the MAO-B enzyme, proceeding by the hydride transfer mechanism.

In our previous EVB studies of MAO enzymes, we have successfully demonstrated that MAO-B lowers the barrier for the oxidative deamination of dopamine by more than nine orders of magnitude compared to the reaction in aqueous solution [36]. Furthermore, this study confirmed the validity of the hydride transfer mechanism of the rate-limiting step of reactions catalyzed by MAO enzymes (Scheme 1). In our earlier study, we demonstrated by quantum mechanical calculations that the hydride transfer mechanism is the most plausible among several proposed mechanisms [37]; this has been further validated by several following studies (see below). The EVB simulation also provided valuable insight into the effect of point mutation on the oxidative deamination of phenylethylamine (PEA) catalyzed by wild-type and mutant I335Y MAO-A [38]. Experimental point mutation studies significantly contributed to the understanding of the specificity and selectivity of MAO enzymes [39], but they still lack explanatory power hidden in molecular interactions governing point mutation effects. Our study demonstrated that I335Y mutation of MAO-A leads to destabilization of the transition state, which is related to attenuation of the quadrupole interaction between the substrate aromatic ring and the active site Phe352 residue. Moreover, mutation allows for an increase in the number of water molecules in the active site, which all together contribute to the reduced catalytic activity of the mutant enzyme by one order of magnitude compared to the wild-type enzyme [38]. Similar studies investigated catalytic degradation of two PEA derivatives by MAO-B [40] and noradrenaline with MAO-A [41], which further supported the hydride abstraction as a rate-limiting step for both MAO isoforms. In addition, extensive in silico mutagenesis explained the functional importance of the “aromatic cage” Tyr444 residue and gave evidence that its hydroxyl group is less important than the benzene moiety for its role in catalysis [41]. Furthermore, the EVB methodology was used to tackle selectivity of both isoforms MAO-A and MAO-B towards adrenaline [42], and to elucidate the reactive step of decomposition of serotonin by MAO-A [43]. In addition to MAO enzymes, an EVB study supported by DFT cluster calculations suggests that the hydride transfer mechanism is involved in the function of the DAO enzyme [44]. 

Apart from EVB methodology, reactions catalyzed by MAO enzymes have been studied by the “traditional” QM/MM approach including DFT methodology in the quantum part [45], or by cluster models treated by DFT [37,46,47]. Furthermore, through a variety of simulation methods, we addressed the mechanism of irreversible inhibition of MAO-B by selegiline and rasagiline, together with their binding into the enzyme’s active site [48], but also focusing on the rate-limiting step by the EVB approach [49]. These studies confirmed that both clinical drugs are mechanism-based inhibitors [50,51], whose activity relies on forming a covalent bond with the enzyme through the rate-limiting hydride abstraction process. Finally, our own multiscale representation of the MAO active site in the electrostatic environment of the solvated enzyme demonstrated the decisive role of electrostatics as the driving force behind the catalytic function of MAO enzymes, as reflected in the barrier lowering, increased charge transfer, and decreased HOMO–LUMO gap [35,52].

The scope of this work is to rationalize and interpret the substantial rate difference between histamine and *N*-methylhistamine for the rate-limiting hydride abstraction during the oxidative deamination reaction catalyzed by MAO-B. As shown in the textbooks [53] and indicated above, *N*-methylhistamine is decomposed by MAO-B at an order-of-magnitude-faster rate than histamine, yet the difference between the substrates is only in a single methyl group. Elucidation of changed reactivity originating from such a small structural difference is *per se* a challenging task, which will be addressed here using EVB methodology. The obtained results provide important insight for several different fields, including enzymology in terms of the rational modification of enzymatic reactions, biotechnology in terms of a rational protein engineering, and pharmacology in terms of the design of improved MAO inhibitors that are clinically used to treat neurodegeneration. 

## 2. Results and Discussion

### 2.1. Reference Reactions in the Gas Phase

**Figure 1:**
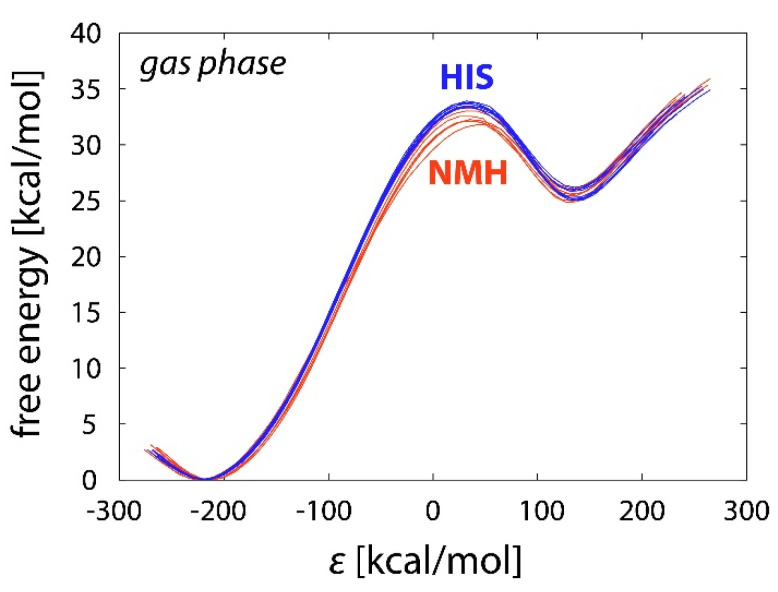
Free energy profiles for the calibrated EVB gas-phase simulations for histamine (HIS/blue) and *N*-methylhistamine (NMH/red), each given in a batch of 10 replicas. *ε* is the generalized reaction coordinate defined by the difference between the potential surfaces of the reactant and product states. The tunable EVB parameters were determined by fitting such that the activation barrier and reaction free energy averaged over the replicas match the values obtained by DFT calculations (Table 1).

The resulting EVB profiles displayed in Figure 1 have, by definition, identical barrier and reaction free energy (averaged over the 10 replicas) as computed by DFT (Table 1). The differences between the two reactions are reflected in the calibrated EVB parameters (Table 1) onto which all the intrinsic effects related to reactivity have been mapped. In the subsequent calculations, in aqueous and enzymatic environments, these parameters are used unchanged, as is the common practice with the EVB approach [54]. 

In agreement with the obvious similarity between HIS and NMH, the difference of 1.06 kcal/mol between the computed barriers for these substrates (Table 1) is rather small. Not surprisingly, geometries of the reactive complex and of the transition-state structure feature only marginal differences between HIS and NMH, thus making it difficult to pinpoint the source of the observed barrier difference to any particular property of the system. While it can be assumed that the differences in the gas phase barriers are governed by a subtle interplay of interactions within and between the reacting molecules, a detailed assessment of these effects would require substantial efforts, representing a challenge that is beyond the scope of this work. 

### 2.2. Reactions in Water

**Figure 2:**
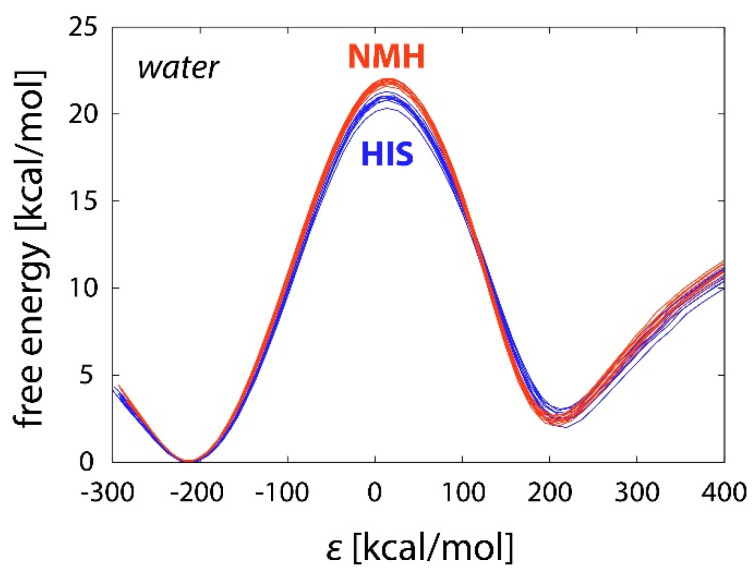
EVB free energy profiles of histamine (HIS/blue) and *N*-methylhistamine (NMH/red) oxidation in aqueous solution, both acquired from 10 simulation replicas. The average barrier and reaction free energy are listed in Table 2. *ε* is the generalized reaction coordinate defined by the difference between the potential surfaces of the reactant and product state. Note that the displayed profiles pertain to the reaction step and do not include the contribution associated with deprotonation of the substrate. The corresponding deprotonation corrections are 3.20 and 2.95 kcal/mol for HIS and NMH, respectively. The total free energy barriers are listed in Table 2.

As the reaction involves significant amount of negative charge transfer from the substrate to the flavin cofactor, accompanied with a charge buildup in the transition-state structure, it can be assumed that the transition state and the product state will have more favorable hydration free energy than the state of reactants in which the reacting entities are neutral, resulting in the lower barrier and a more exergonic reaction free energy, as compared with the gas phase. This is clearly reflected in the corresponding EVB profiles displayed in Figure 2. 

**Figure 3:**
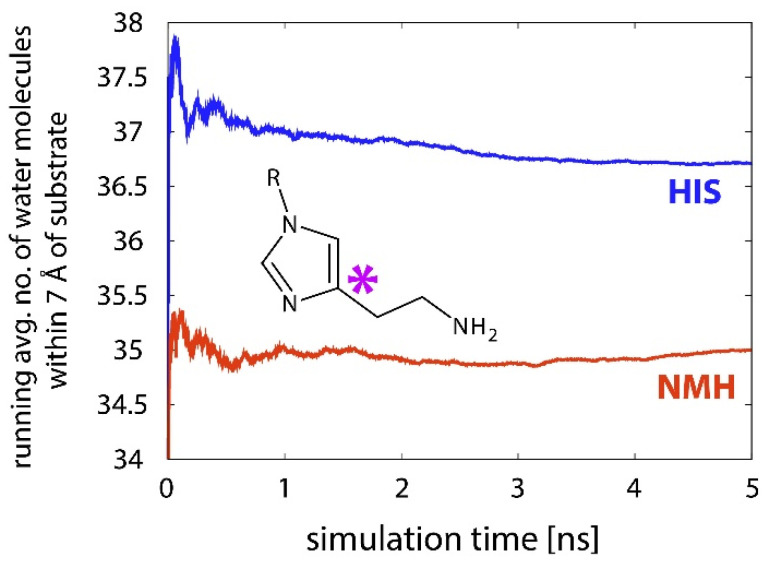
Running average number of water molecules within 7 Å of the C4 atom of the imidazole ring (marked with purple star) of histamine (HIS/blue) and *N*-methylhistamine (NMH/red) computed over reaction simulation.

The barriers are by around 12 kcal/mol lower than in the gas phase, which converts into a boost of about nine orders of magnitude in the reaction rate. Also, for the same reason, free energy of the simulated reaction step shifts from significantly endergonic (~25 kcal/mol) to just slightly endergonic (~3 kcal/mol). However, in the context of the present study, perhaps the most significant difference from the gas phase reaction is in the changed order of the barriers: Unlike in the gas phase, reaction involving HIS is linked with a 0.63 kcal/mol lower barrier than with NMH—after deprotonation correction, the barriers amount to 24.12 vs. 24.75 kcal/mol, respectively (see Figure 2 and Table 2). A plausible explanation is that HIS is better solvated with water molecules than NMH, whose ring methyl group hinders solvation. Indeed, inspection of simulation trajectories reveals that, on average, about two more water molecules can be found in the vicinity of HIS than around NMH (Figure 3). This feature is persistent over the entire course of reaction. Since the polarity of the reacting moiety steadily increases during the reaction (for NMH, the dipole moment elevates from 9.4 D in the state of reactants to 13.4 D in the transition state, whereas for HIS, the dipole moment increases from 8.9 D to 12.8 D), the susceptibility of the free energy to variations in solvation is larger in the transition state; hence, poorer solvation of NMH results in the higher barrier.

### 2.3. Reactions in the Enzyme

**Figure 4:**
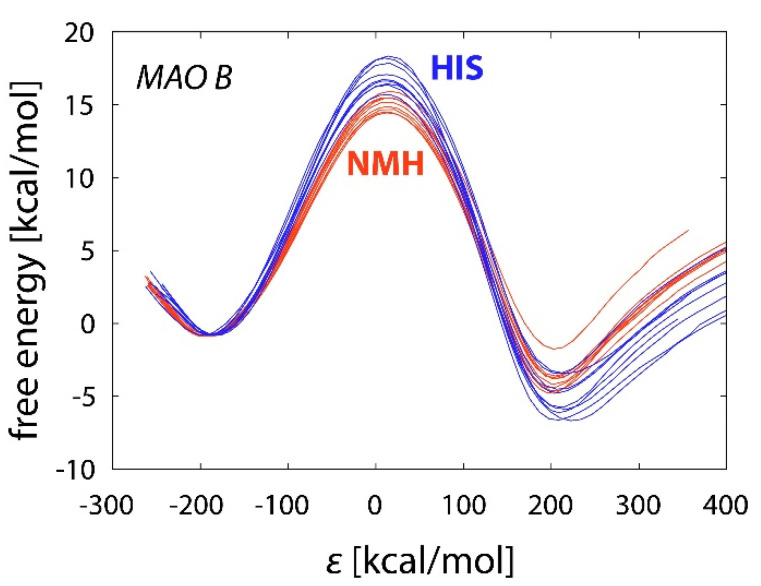
EVB free energy profiles for histamine (HIS/blue) and N-methylhistamine (NMH/red) oxidation in monoamine oxidase B (MAO-B), both acquired from 10 simulation replicas. The average barrier and reaction free energy are listed in Table 2. *ε* is the generalized reaction coordinate defined by the difference between the potential surfaces of the reactant and product state. Note that the displayed profiles pertain to the reaction step and do not include the contribution associated with deprotonation of the substrate. The corresponding deprotonation corrections are 3.20 and 2.95 kcal/mol for HIS and NMH, respectively. The total free energy barriers are listed in Table 2.

The complex enzymatic environment provides catalytic effect optimized by millions of years of evolution; hence, it can be expected that the reaction barriers in the enzyme are further reduced with respect to a disordered polar solvent. Indeed, the computed barriers for HIS and NMH oxidation are about 5 kcal/mol lower than in water, namely (after deprotonation correction) 21.04 and 18.95 kcal/mol, respectively (Figure 4 and Table 2). 

**Figure 5:**
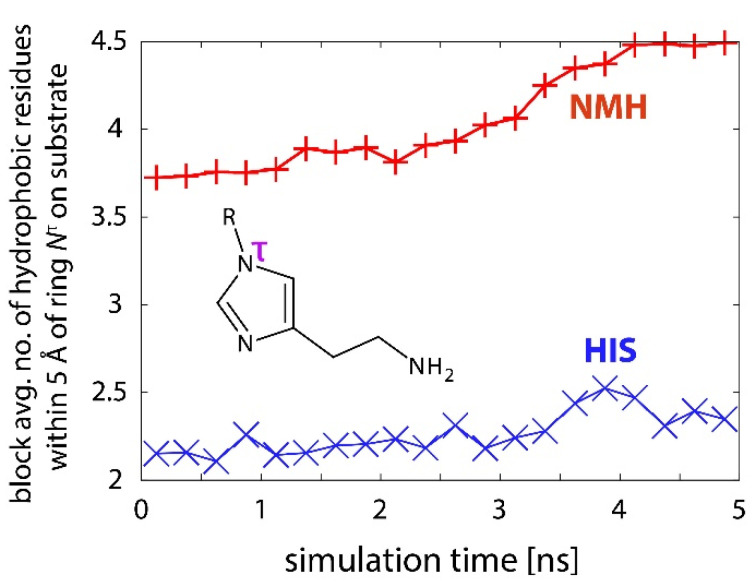
Average number (in 0.25 ns blocks) of hydrophobic residues within 5 Å of the common ring nitrogen atom (N^τ^) of both substrates (histamine: HIS/blue; *N*-methylhistamine: NMH/red) during simulation of the reaction.

**Figure 6:**
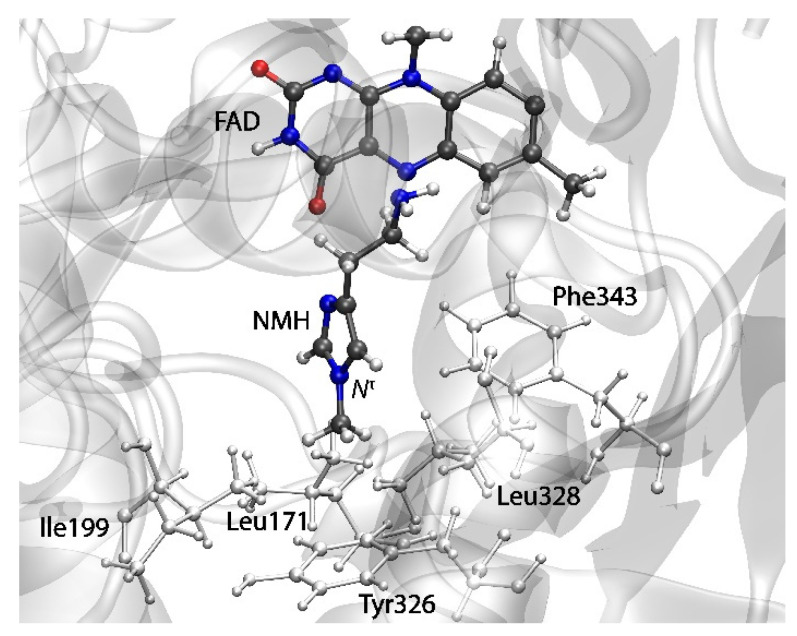
*N*-methylhistamine (NMH) in the active site of monoamine oxidase B during reaction simulation, with surrounding hydrophobic residues and Tyr326 aromatic residue indicated.

In contrast to aqueous solution, the free energy barrier for NMH oxidation is again lower than that of HIS (as in the gas phase). A possible explanation can be derived from hydrophobic interactions despite these interactions are properly defined only for aqueous solution, but not for complex environments, such as enzyme active sites [55]. The lower barrier for NMH can be rationalized by the fact that the active site of MAO-B is rich with amino acids with hydrophobic and aromatic side chains, which has been the subject of a separate investigation of preorganized electrostatics in MAO-A and MAO-B [56]. Since NMH is larger and more hydrophobic than HIS, it is capable of establishing a larger number of favorable dispersion interactions with nonpolar residues as well as electrostatic interactions with aromatic moieties at the active site than HIS (nearly two more on average, see Figure 5), thus the methyl group of NMH acts as an anchor holding the molecule in a proper orientation for the reaction (Figure 6), as demonstrated earlier [19]. Likewise, it has been documented that dopamine and phenylethylamine are better MAO substrates than NMH [13,39], which can be attributed to the fact that they are even more hydrophobic. To support that, the ChEMBL database [57] lists the following partition coefficient logP values for dopamine (0.60), phenylethylamine (1.19), NMH (–0.08), and HIS (–0.09), which clearly indicate a much higher hydrophobic character of dopamine and phenylethylamine over NMH and HIS. In principle, this provides a plausible explanation for the better binding of hydrophobic substrates to the MAO active site; however, for the elucidation of their reactivity, additional evidence is required because reactivity is, to a large extent, independent of binding. The reaction trajectories offer a reasonable explanation of the lowered barrier for NMH oxidation relative to HIS.

The number of favorable hydrophobic contacts between the substrate and surrounding side chains slightly increases during the reaction (Figure 5). This effect is more pronounced with NMH than with HIS. Therefore, stabilization due to hydrophobic interactions is larger for the transition state structures, and at the same time, it is larger for NMH than for HIS, contributing to the more pronounced barrier-lowering with the former. 

Another feature possibly contributing to the lower barrier for NMH decomposition relative to HIS originates from hydration effects caused by a small number of water molecules present in the enzyme active site. As already demonstrated for aqueous solution, NMH is less solvated by water molecules than HIS; the number of water molecules around NMH is, on average, about one less than around HIS during the reaction in the enzyme. In contrast to the aqueous environment, in the enzyme active site, poorer hydration is reflected in increased catalytic effect because fewer water molecules provide less dielectric shielding of the electrostatic interactions between the reacting moiety and the enzymatic surroundings. As demonstrated in a number of cases, including MAO-A and MAO-B [35,52], these interactions represent the main source of catalysis. The same effect has been observed in our recent investigation of enzyme point mutation effect of phenylethylamine oxidation by MAO-A [38]. Overall, we rationalize that the cumulated hydrophobic and hydration effects contribute to the perceivably lower barrier for NMH oxidation by MAO-B, as compared to HIS. 

Table 2 lists the EVB free energy barriers for all three considered environments. The catalytic effect exhibited by water and, particularly, the enzymatic environment is clearly reflected in significantly lower barriers, as compared to the gas phase. The free energy barriers computed for HIS and NMH in MAO-B are about 1–2 kcal/mol higher than ones obtained by experimental kinetic studies [13]; however, they are still in very reasonable agreement with the experimental values. Still, we must emphasize a success of the employed EVB methodology in estimating relative differences among substrates, where experimentally determined ΔΔ*G*^‡^ = 1.37 kcal/mol is nicely reproduced by the calculated value of 2.09 kcal/mol, which gives strong support for the postulated hydride abstraction mechanism. While the free energy of deprotonation has been included in the present treatment, the correction relies on p*K*_a_ values measured in the aqueous solution [58], thereby assuming that the p*K*_a_ value of either substrate remains unchanged when passing from aqueous solution to the enzyme active site. Recent evidence that p*K*_a_ of the chemically similar dopamine substrate is only marginally different between aqueous environment and the MAO-B active site [56,59] supports our assumption. As a challenge, the present treatment could be improved by performing the state-of-the-art p*K*_a_ calculations [60,61] of HIS and NMH within the active site of MAO-B, which is a complex task and beyond the scope of this work. 

Another aspect worthy of discussion is the reaction sampling quality, reflected in deviations between the free energy profiles, as reflected in standard errors of the mean (SEM) between the simulation replicas (Table 2). This statistical parameter is indicative of the sensitivity of the average barrier to variations between the replicas, which are due to the limitations of the model (e.g., limited phase space sampling). Ideally, for the completely sampled reaction phase space, the free energy profile should be independent of the initial conditions, and all the replicas would yield identical profiles [62]. However, due to the limited simulation time, the profiles remain different from one another, indicating incomplete sampling. In agreement with the complexity of the model, the deviations are larger in enzyme simulations than in the gas phase or in water. Nevertheless, SEM is in all cases significantly smaller than the compared barriers (e.g., the barrier difference between NMH and HIS is just over 2 kcal/mol, whereas the corresponding SEM values do not exceed 0.3 kcal/mol). Therefore, we conclude on a qualitative level that, despite limitations and inherent inaccuracies of the model, the reported barrier differences between NMH and HIS are statistically significant, and the herein reported results appear to be valid and illustrative.

## 3. Materials and Methods

The core part of the present treatment is the calculation of free energy profiles (including free energy of activation and reaction free energy) of the rate-limiting hydride abstraction step of the oxidative deamination of HIS and NMH catalyzed by MAO-B, by using the EVB methodology. The treatment consists of a series of classical simulations employing a two-state force field corresponding to reactants and products. While the reactants are represented by a neutral substrate (HIS or NMH) docked in the enzyme active site, we took for the product state the complex with the substrate alpha C—H hydrogen transferred to the N5 atom of the flavin cofactor as a hydride anion, as described earlier by DFT, ONIOM (our own n-layered integrated molecular orbital and molecular mechanics [63]) and QM/MM mechanistic studies (Scheme 1) [37,46,64,65]. The simulation of the reaction was facilitated by the free energy perturbation (FEP) protocol, as implemented in the program package *Q v. 5* [66]. We used the *qdyn5* module of the package as the main simulation engine. 

**Figure 7:**
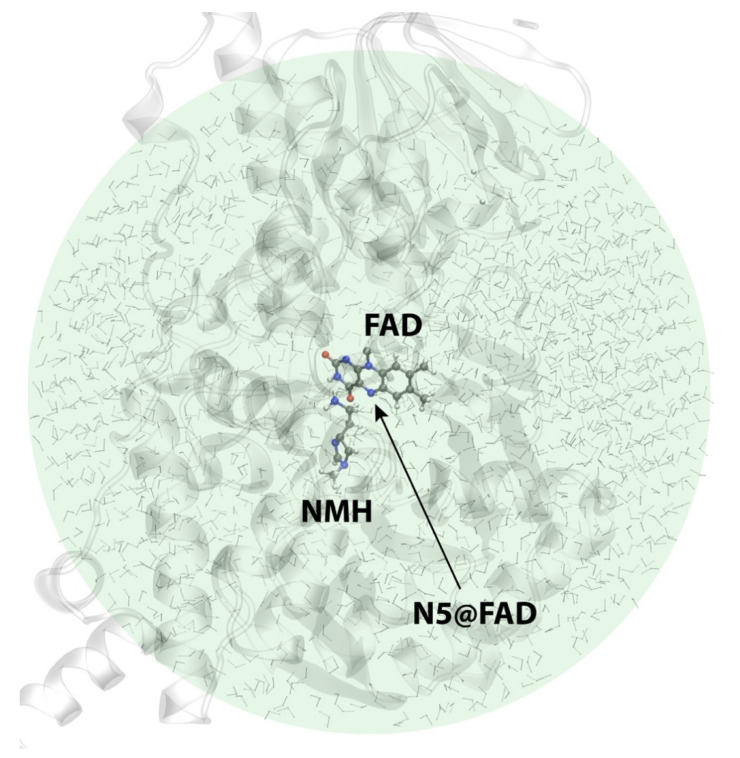
Structure of the solvated monoamine oxidase B (MAO-B) with the reacting *N*-methylhistamine (NMH) and flavin adenine dinucleotide (FAD) prosthetic group in a spherical simulation cell centered at the N5 atom of FAD.

The reaction model in the enzyme was built on the basis of the crystal structure of MAO-B acquired from the Protein Data Bank (codename 2XFN) [67]. The model included one subunit of MAO-B enclosed in a spherical simulation cell with a radius of 30 Å centered at the reactive N5 atom of the flavin cofactor and contained 1687 water molecules (see Figure 7). The docking of HIS and NMH substrate molecules was assisted by our recent work on the selectivity of MAO-B towards these two molecules employing molecular dynamics simulations and quantum calculations [19]. The simulations were built around the optimized potentials for liquid simulations (all atom) (OPLS–AA) force field [68,69] for the protein and substrate and the transferable intermolecular potential with 3 points (TIP3P) model of water. Parameters for the FAD cofactor covalently bound to the Cys397 residue were taken from our previous study [36]. Atomic charges of the reacting species (a substrate molecule and the flavin cofactor) were computed at the HF/6-31G(d) level of theory by the RESP scheme using the Antechamber [70] module of the AmberTools15 suite of programs [71]. The relaxation and equilibration procedure included 2.2 ns of simulation. The FEP protocol consisted of 51 steps gradually converting the force field of reactants into one of the products, by adding at each successive step 2% character of the products to the model Hamiltonian and simultaneously removing the same amount of share of reactants from it. The duration of each FEP step was 100 ps, totaling to 5.1 nanoseconds of the reaction simulation. In order to improve the quality of sampling of the reaction pathway, FEP simulations were performed in batches of 10 replicas generated by randomization of velocities at the end of stage of equilibration and by running a short relaxation of 100 ps, resulting in 10 distinct pathways and corresponding free energy profiles. The final result is given as an average of these replicas and deviation between them is also considered by means of computing the standard error of the mean of the replicas, defined as standard deviation (of the 10 replicas) divided by the square root of the sample size (10). To avoid divergent and noisy profiles associated with the limited simulation length [62], weak position restraints with the harmonic force constant of 0.5 kcal/(mol Å^2^) were used on the reacting atoms, as has been common in related studies [36,38,41,72]. In addition to simulations in the enzyme, the same reaction was modeled in the explicit aqueous environment and in the gas phase by the identical protocol, including the equilibration, FEP simulations and calculation of EVB free energy profiles.

The EVB free energy profiles were acquired *a posteriori* by analyzing the data produced by FEP simulations. For this purpose, we used the *qfep5* program of the *Q* package. Empirical parameters required for the EVB treatment were obtained from the equivalent reaction simulated in the gas phase by fitting to the activation and reaction free energy (Table 1) computed at the M06-2X/6-31+G(d,p) level by Gaussian09 [73] following standard optimization procedures. Vibrational zero-point energy and thermal free energy terms were included by means of harmonic vibrational analysis carried out on optimized structures. 

Monoamine substrates react in neutral (un-ionized) forms, as suggested both by experiment and calculations [42,59,74,75,76], whereas at physiological pH of 7.4, both HIS and NMH are predominantly present as monocationic forms protonated at their ethylamino groups, as reflected in their aqueous p*K*_a_ values of 9.75 and 9.57, respectively [58]. This requires the computed free energy barriers to be corrected for the free energy associated with deprotonation to neutral forms (Δ*G*_deprot_) according to the formula $\Delta G_{deprot}=2.303\;k_{B}T\;\left(pK_{a}-pH\right)$, where *k*_B_ is Boltzmann constant, and *T* is the absolute temperature. At physiological pH of 7.4 and 300 K, the deprotonation corrections for HIS and NMH are 3.20 and 2.95 kcal/mol, respectively. These values were added to the EVB barriers computed for the reaction in the enzyme as well as in aqueous solution, all of which involved the neutral amino group on HIS and NMH, which is a valid approximation given the demonstrated independence of the matching substrate p*K*_a_ values on-going from pure water onto the MAO-B active site [59]. 

All essential simulation parameters, starting geometries, and transition-state structures are deposited as Supplementary Materials (Figures S1–S2, Tables S1–S11). For a more detailed description of the employed methodology, the reader is referred to our recent study of enzyme point mutation effect on kinetics of decomposition of phenylethylamine catalyzed by MAO-A [38].

## 4. Conclusions

Histamine degradation in the human central nervous system is initiated by a methylation reaction. The resulting *N*^τ^-methylated histamine is further metabolized by MAO-B, while histamine itself, under normal conditions, is not a substrate for this enzyme in vivo. This transformation through methylation is possibly essential, not only for the enhanced kinetics, but also for other reasons. Namely, direct oxidation of histamine by MAO-B would produce imidazole acetic acid, which is a GABA receptor agonist, and as such would lead to altered neurochemical and behavioral properties expressed through sedative effects, anxiolytic, anticonvulsant, and muscle relaxant effects [77,78,79,80]. Our study fairly reproduces and explains the observed MAO-B enzyme’s one order-of-magnitude preference for the *N*-methylated form of histamine [13] and gives valuable insight into this particular way of not only regulating histamine concentration in the brain, but also evading adverse metabolites. In other words, our research suggests that MAO-B can directly metabolize histamine in vivo, but only under conditions when HNMT is inhibited or deficient. Administration of HNMT enzyme inhibitors, therefore, could lead to alternative histamine oxidation through MAO-B enzyme activity, producing imidazole acetic acid. This is consistent with the in vitro kinetic study demonstrating the capability of MAO-B to decompose histamine, but with poorer catalytic performance relative to *N*-methylhistamine [13]. 

The experimental barrier difference between histamine and *N*-methylhistamine is only 1.37 kcal/mol and is well-reproduced by the present treatment, yielding the difference of 2.09 kcal/mol, while tying both calculated values with experiments in the absolute sense as well. In this respect, the presented results offer convincing evidence in favor of the feasibility of the hydride transfer mechanism for the MAO catalytic activity. From the mechanistic point of view, results presented here together with our previous study [44] show that the rate-limiting steps for the *N*-methylhistamine degradation by MAO-B and histamine degradation by DAO enzymes proceed through a direct hydride transfer from substrate to the corresponding FAD and TPQ cofactors, respectively, and this notion is independent of the histamine degradation pathway. This is reasonable since the introduced imidazole *N*-methyl group is located far away from the reacting ethylamino moiety that gets oxidized, thereby unlikely changing the precise mechanism of the catalytic transformation. Since the applied EVB approach gives a detailed, atomistic insight into interactions relevant for enzyme-catalyzed reactions, a thorough explanation of the source of selectivity has been delivered from the present simulations, which confirms the hydrophobic nature of the MAO-B active site and results in an improved understanding of the mechanism of histamine inactivation by the MAO-B enzyme. 

## Figures and Tables

**Table 1:** M06-2X/6-31+G(d,p) calculated barriers (Δ*G*^‡^) and reaction free energies (Δ*G*_R_) for the rate-limiting hydride abstraction step of histamine (HIS) and *N*-methylhistamine (NMH) oxidation by flavin in the gas phase, together with the calibrated EVB parameters acquired from gas-phase FEP simulations by fitting to the listed DFT values.

Substrate	DFT Calculations		Calibrated EVB Parameters	
	Δ*G*^‡^ [kcal/mol]	Δ*G*_R_ [kcal/mol]	*H*_12_ [kcal/mol]	α_2_^0^ [kcal/mol]
HIS	33.52	25.84	72.81	88.08
NMH	32.46	26.30	74.94	98.50

**Table 2:** Free energy barriers and their standard errors of the mean (in parentheses) for the rate-limiting step of histamine (HIS) and *N*-methylhistamine (NMH) oxidation by flavin in the gas phase, water, and enzyme environment. For the reaction in monoamine oxidase B (MAO-B), experimental values are also given [13]. All values are given in kcal/mol. Note that the computed barriers in water and in MAO-B include the correction associated with deprotonation of the substrate.

Substrate	Gas Phase	Water	MAO-B	Exp.
**HIS**	33.52 (0.06)	24.12 (0.08)	21.04 (0.29)	19.26
**NMH**	32.46 (0.17)	24.75 (0.05)	18.95 (0.16)	17.89

## Data Availability

The relevant simulation data is posted as Supplementary Materials.

## Supplementary Materials

The following are available online at https://www.mdpi.com/article/10.3390/ijms23031910/s1.
