# Supplementary material for: Why Monoamine Oxidase B Preferably Metabolizes N-Methylhistamine over Histamine: Evidence from the Multiscale Simulation of the Rate-Limiting Step
Source: Int J Mol Sci. 2022 Feb 8;23(3):1910. doi: 10.3390/ijms23031910 (PMC8836602; doi:10.3390/ijms23031910)
Supplement: Supplementary file 1 [file ijms-23-01910-s001.zip › Supplementary Materials.pdf]

Supplementary Materials

Why Monoamine Oxidase B Preferably Metabolizes *N*-methylhistamine over Histamine: Evidence from the Multiscale Simulation of the Rate Limiting Step

Aleksandra Maršavelski,[a, b, d] Janez Mavri,[d] Robert Vianello [a, c,]* and Jernej Stare [d,]*

[a] Computational Organic Chemistry and Biochemistry Group, Ruđer Bošković Institute, Bijenička 54, HR-10000 Zagreb, Croatia (affiliation at the time of research)
[b] Department of Chemistry, Faculty of Science, University of Zagreb, Horvatovac 102a, HR-10000 Zagreb, Croatia (present affiliation)
[c] Laboratory for the Computational Design and Synthesis of Functional Materials, Ruđer Bošković Institute, Bijenička 54, HR-10000 Zagreb, Croatia (present affiliation)
[d] Theory Department, National Institute of Chemistry, Hajdrihova 19, SI-1000 Ljubljana, Slovenia
* Corresponding authors, e-mail: robert.vianello@irb.hr (R.V.), jernej.stare@ki.si (J.S.)

Table of Contents

Figure S1. Valence bond states and atom numbering used in this work to describe the hydride transfer from histamine to FAD of MAO B. 3

Figure S2. Valence bond states and atom numbering used in this work to describe the hydride transfer from N-methylhistamine to FAD of MAO B. 4

Table S1. List of ionized residues, as well as the protonation states of histidines, in the EVB simulations 5

Table S2. Nonbonding parameters used to describe the two EVB states during the direct hydride transfer in the oxidative deamination of histamine by MAO-B 6

Table S3. Bonding parameters used to describe the two EVB states during the direct hydride transfer step of the oxidative deamination of histamine by MAO-B 7

Table S4. Angle parameters used to describe the two EVB states for the hydride transfer step of the oxidative deamination of histamine by MAO-B 8

Table S5. Torsion parameters used to describe the two EVB states for the hydride transfer step of the oxidative deamination of histamine by MAO-B. 9

Table S6. Improper torsion types used to describe the two EVB states for the hydride transfer step of the oxidative deamination of histamine by MAO-B 10

Table S7. Nonbonding parameters used to describe the two EVB states during the direct hydride transfer in the oxidative deamination of N-methylhistamine by MAO-B. 11

Table S8. Bonding parameters used to describe the two EVB states during the direct hydride transfer step of the oxidative deamination of N-methylhistamine by MAO-B 12

Table S9. Angle parameters used to describe the two EVB states for the direct hydride transfer step of the oxidative deamination of N-methylhistamine by MAO-B 13

Table S10. Torsion parameters used to describe the two EVB states for the direct hydride transfer step of the oxidative deamination of N-methylhistamine by MAO-B 14

Table S11. Improper torsion types used to describe the two EVB states for the direct hydride transfer step of the oxidative deamination of N-methylhistamine by MAO-B. 15

Transition state histamine (TS) 16

Transition state N-methylhistamine (TS) 17

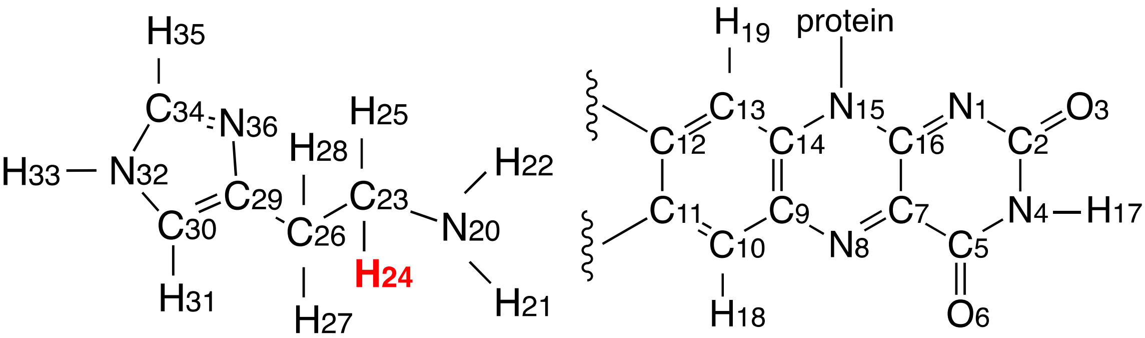

State I

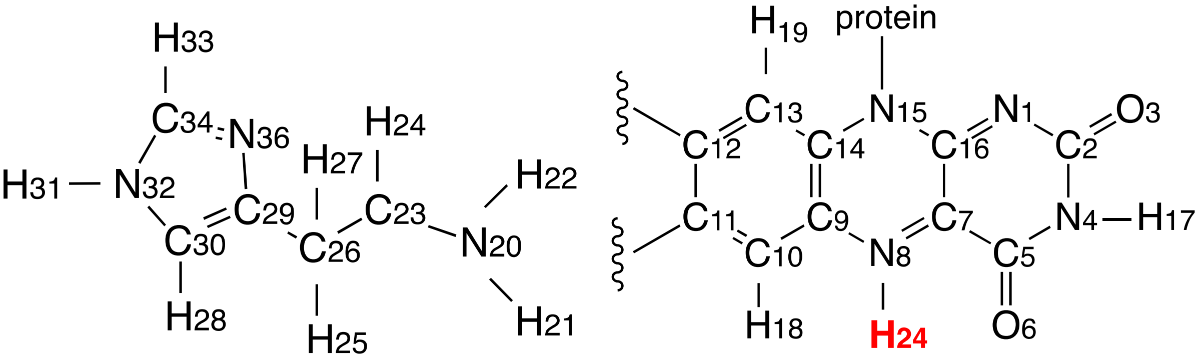

State II

Figure S1. Valence bond states and atom numbering used in this work to describe the hydride transfer from histamine to FAD of MAO B. States I and II correspond to the reactant and intermediate states of the catalytic cycle, respectively. The hydride being transferred is labeled in red.

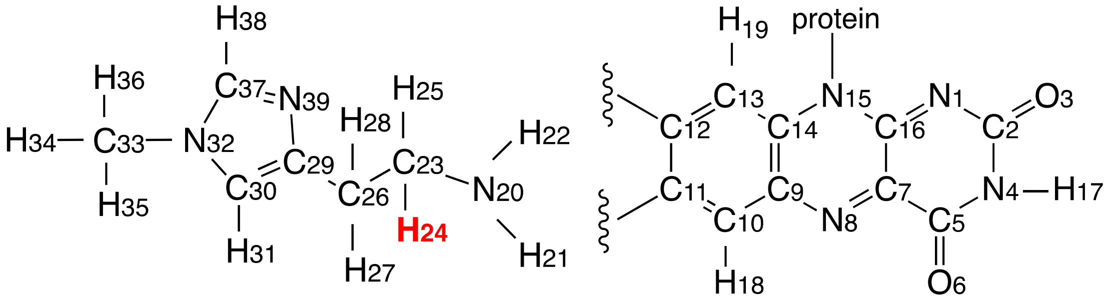

State I

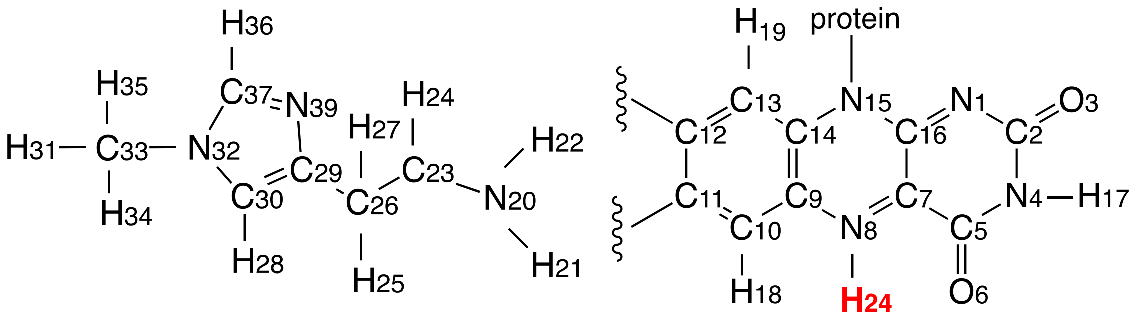

State II

Figure S2. Valence bond states and atom numbering used in this work to describe the hydride transfer from N-methylhistamine to FAD of MAO B. States I and II correspond to the reactant and intermediate states of the catalytic cycle, respectively. The hydride being transferred is labeled in red.

Table S1. List of ionized residues, as well as the protonation states of histidines, in the EVB simulations. The remaining residues were kept in their neutral forms, as they were located outside the 30 Å explicit simulation sphere centered at the reactive nitrogen atom of the flavin cofactor, as described in the main text (see also ref. 38 cited therein).

Residue	Residue number
Asp	37, 55, 123, 132, 144, 223, 318, 329, 330, 471
Glu	34, 74, 78, 84, 86, 176, 179, 207, 219, 232, 320, 321, 334, 358, 384, 385, 390, 391, 427, 437, 441, 444, 466, 468,
Lys	21, 50, 52, 73, 81, 190, 209, 271, 296, 332, 351, 348, 357, 363, 386
Arg	36, 38, 42, 47, 67, 70, 197, 208, 220, 282, 307, 350, 354, 360, 415, 445, 448
His-δ	/
His-ε	24, 90, 91, 115, 178, 252, 273, 347, 382, 431, 452, 485

Table S2. Nonbonding parameters used to describe the two EVB states during the direct hydride transfer in the oxidative deamination of histamine by MAO-B.[a]

Atom number	State I			State II		
	A_i	B_i	q_i	A_i	B_i	q_i
1	885.43	27.02	-0.7443	971.75	28.31	-0.8619
2	1802.24	34.18	1.0090	1802.24	34.18	0.9920
3	616.44	23.77	-0.6117	616.44	23.77	-0.7546
4	971.75	28.31	-0.7738	971.75	28.31	-0.7680
5	1802.24	34.18	0.7294	1039.88	24.25	0.7563
6	616.44	23.77	-0.5553	976.93	31.26	-0.7333
7	1059.13	23.67	0.2576	1039.88	24.25	-0.4382
8	971.75	28.31	-0.5511	1064.97	29.63	-0.4260
9	1059.13	23.67	0.4846	1059.13	23.67	0.2654
10	1059.13	23.67	-0.3610	1059.13	23.67	-0.5285
11	1059.13	23.67	0.1155	1059.13	23.67	0.2023
12	1059.13	23.67	0.1463	1059.13	23.67	0.0387
13	1059.13	23.67	-0.2837	1059.13	23.67	-0.3640
14	1059.13	23.67	-0.1662	1059.13	23.67	0.0233
15	1064.97	29.63	0.0996	971.75	28.31	-0.0233
16	58.02	5.09	0.3831	1039.88	24.25	0.5280
17	0.00	0.00	0.4042	0.00	0.00	0.3553
18	69.58	4.91	0.2236	69.58	4.91	0.2061
19	69.58	4.91	0.1942	69.58	4.91	0.1982
20	1064.9718	29.6344	-1.1151	971.7502	28.3077	-0.6128
21	0.0054	0.0433	0.3880	0.0054	0.0433	0.4160
22	0.0054	0.0433	0.3880	0.0054	0.0433	0.4160
23	944.5180	22.0296	0.7774	1802.2385	34.1758	0.4763
24	59.8020	3.8273	-0.1011	0.00	0.00	0.3322
25	59.8020	3.8273	-0.1011	69.5797	4.9095	0.1311
26	944.5180	22.0296	-0.6418	944.5180	22.0296	-0.4045
27	84.5728	5.4127	0.1318	84.5728	5.4127	0.1708
28	84.5728	5.4127	0.1318	84.5728	5.4127	0.1708
29	1059.1297	23.6736	0.5435	1059.1297	23.6736	0.4185
30	1059.1297	23.6736	-0.3786	1059.1297	23.6736	-0.3488
31	69.5797	4.9095	0.2198	69.5797	4.9095	0.2564
32	971.7502	28.3077	-0.3306	971.7502	28.3077	-0.2222
33	0.0054	0.0433	0.3499	0.0054	0.0433	0.3610
34	1059.1297	23.6736	0.2303	1059.1297	23.6736	0.1491
35	69.5797	4.9095	0.1145	69.5797	4.9095	0.1769
36	971.7502	28.3077	-0.6068	971.7502	28.3077	-0.5545

[a] The atom numbering corresponding to valence bond states shown in Figure S1, with States I and II corresponding to the reactant and product states respectively. Van der Waals interactions were described by the 12-6 Lennard Jones potential, where A_i and B_i are single-atom (square-rooted) parameters in units of $kcal^{1/2}\cdot mol^{-1/2}\cdot Å^{-6}$ and $kcal^{1/2}\cdot mol^{-1/2}\cdot Å^{-3}$, respectively, while q_i is the partial atomic charge.

Table S3. Bonding parameters used to describe the two EVB states during the direct hydride transfer step of the oxidative deamination of histamine by MAO-B.[a]

**Atom number**		**State I**		**State II**	
**#1**	**#2**	f_c	r_0	f_c	r_0
1	2	1000.00	1.261	914.00	1.358
1	16	1000.00	1.261	966.00	1.339
2	4	980.00	1.335	836.00	1.388
4	5	980.00	1.335	896.00	1.365
5	6	1140.00	1.229	900.00	1.370
5	7	938.00	1.400	1098.00	1.340
7	8	914.00	1.358	962.00	1.340
7	16	800.00	1.490	854.00	1.433
8	9	966.00	1.339	962.00	1.340
20	21	868.00	1.0100	980.00	1.0150
20	22	868.00	1.0100	980.00	1.0150
20	23	764.00	1.4480	1270.00	1.2860
23	25	680.00	1.0900	770.00	1.0830
23	26	536.00	1.5290	580.00	1.4880

		D_e	a	r_0	D_e	a	r_0
8	24	*n.a.*	*n.a.*	*n.a.*	100.60	2.000	1.100
23	24	102.76	2.000	1.100	*n.a.*	*n.a.*	*n.a.*

[a] The atom numbering corresponds to the atoms displayed in Figure S1, with States I and II corresponding to the reactant and intermediate states, respectively. Bonds were described with either a harmonic potential of the form $E_{bond} = ½ \cdot f_c\,(r - r_0)^2$, or in the case of forming or breaking bonds, with a Morse potential of the form $E_{bond} = D_e \cdot (e^{-2a\cdot(r-r_0)} - 2e^{-a\cdot(r-r_0)})$. All f_c and D_e values are given in kcal·mol^{-1}, and r_0 values in Å.

Table S4. Angle parameters used to describe the two EVB states for the hydride transfer step of the oxidative deamination of histamine by MAO-B.[a]

**Atom number**			**State I**		**State II**	
**#1**	**#2**	**#3**	f_c	θ	f_c	θ
1	2	3	160.00	120.600	160.00	122.500
1	2	4	140.00	114.200	140.00	118.600
1	16	15	140.00	124.100	140.00	119.300
2	1	16	100.00	120.500	140.00	120.500
2	4	5	140.00	126.400	140.00	121.600
2	4	17	70.00	119.800	70.00	116.800
3	2	4	160.00	122.900	160.00	120.600
4	5	6	160.00	122.900	160.00	120.600
4	5	7	140.00	116.600	140.00	121.200
5	4	17	70.00	119.800	70.00	119.200
5	7	8	160.00	123.000	140.00	121.200
5	7	16	160.00	117.200	170.00	170.00
6	5	7	160.00	121.400	140.00	123.00
7	8	9	140.00	120.500	100.00	109.500
7	8	24	*n.a.*	*n.a.*	70.00	111.00
8	7	16	160.00	123.00	140.00	120.100
8	9	10	140.00	124.00	140.00	120.100
8	9	14	140.00	124.00	140.00	120.100
9	8	24	*n.a.*	*n.a.*	70.00	111.00
20	23	24	70.00	109.500	*n.a.*	*n.a.*
20	23	25	70.00	109.500	90.00	118.0390
20	23	26	112.40	109.4700	140.00	120.6520
21	20	22	87.20	106.4000	60.00	117.8320
21	20	23	70.00	109.5000	90.00	118.8950
22	20	23	70.00	109.5000	90.00	118.8950
23	26	27	75.00	110.7000	90.00	109.3340
23	26	28	75.00	110.7000	90.00	109.3340
23	26	29	140.00	112.6240	140.00	112.3600
24	23	25	66.00	107.800	*n.a.*	*n.a.*
24	23	26	75.00	110.700	*n.a.*	*n.a.*
25	23	26	75.00	110.700	70.00	121.9150

[a] The atom numbering corresponding to the atom numbering used here are displayed in Figure S1, with States I and II corresponding to reactant and product states, respectively. The bending motion was described with a harmonic potential of the form $E_{angle} = f_c \cdot (\theta - \theta_0)^2$, where f_c is given in kcal·mol^{-1} and θ in °.

Table S5. Torsion parameters used to describe the two EVB states for the hydride transfer step of the oxidative deamination of histamine by MAO-B.[a]

Atom number				State I			State II		
#1	#2	#3	#4	V_1	V_2	V_3	V_1	V_2	V_3
1	2	4	5	0.000	3.968	0.000	0.000	2.325	0.000
1	2	4	17	0.000	1.015	0.000	0.000	2.325	0.000
5	7	16	1	0.000	0.839	0.000	0.000	7.000	0.000
8	7	16	1	0.000	3.500	0.000	0.000	7.000	0.000
14	15	16	1	0.000	1.826	0.000	0.000	3.968	0.000
2	1	16	7	-1.183	0.456	-0.425	0.000	3.625	0.000
2	1	16	15	0.000	1.826	0.000	0.000	3.625	0.000
2	4	5	6	0.000	3.045	0.000	0.000	3.625	0.000
2	4	5	7	1.150	3.045	0.000	0.000	3.625	0.000
16	1	2	4	0.000	1.826	0.000	0.000	5.000	0.000
4	5	7	8	0.000	3.500	0.000	0.000	7.000	0.000
4	5	7	16	0.000	0.550	0.000	0.000	7.000	0.000
5	7	8	9	0.000	7.000	0.000	-3.791	1.715	1.599
5	7	8	24	*n.a.*	*n.a.*	*n.a.*	0.000	1.015	0.000
5	7	16	15	0.000	0.839	0.000	0.000	7.000	0.000
17	4	5	6	0.000	2.450	0.000	0.000	3.625	0.000
6	5	7	8	0.000	0.410	0.000	0.000	7.000	0.000
6	5	7	16	0.000	1.050	0.000	0.000	7.000	0.000
17	4	5	7	0.000	2.450	0.000	0.000	1.525	0.000
7	8	9	10	0.000	3.625	0.000	-3.791	1.715	1.599
7	8	9	14	0.000	3.625	0.000	-3.791	1.715	1.599
8	7	16	15	0.0000	3.500	0.000	0.000	7.000	0.000
16	7	8	9	0.000	7.000	0.000	-3.791	1.715	1.599
24	8	9	10	*n.a.*	*n.a.*	*n.a.*	0.000	1.015	0.000
24	8	9	14	*n.a.*	*n.a.*	*n.a.*	0.000	1.015	0.000
16	7	8	24	*n.a.*	*n.a.*	*n.a.*	0.000	1.015	0.000
20	23	26	27	0.1500	0	0	0	0	0
20	23	26	28	0.1500	0	0	0	0	0
20	23	26	29	0.8375	0.3220	0.7060	0	0	0
21	20	23	24	0.2000	0	0	*n.a.*	*n.a.*	*n.a.*
21	20	23	25	0.2000	0	0	2.000	0	0
21	20	23	26	-0.0595	0.0885	0.1175	2.000	0	0
22	20	23	24	0.2000	0	0	*n.a.*	*n.a.*	*n.a.*
22	20	23	25	0.2000	0	0	2.000	0	0
22	20	23	26	-0.0595	0.0885	0.1175	2.000	0	0
23	26	29	30	0.0415	0	0	0.1805	0	0
23	26	29	36	0.2930	-1.1970	0.6090	0.0430	0	0
24	23	26	27	0.1500	0	0	*n.a.*	*n.a.*	*n.a.*
24	23	26	28	0.1500	0	0	*n.a.*	*n.a.*	*n.a.*
24	23	26	29	0.4320	0	0	*n.a.*	*n.a.*	*n.a.*
25	23	26	27	0.1500	0	0	0.3310	0	0
25	23	26	28	0.1500	0	0	0.3310	0	0
25	23	26	29	0.4320	0	0	0	0	0

[a]The atom numbering corresponds to the atom numbers used in Figure S1, with States I and II corresponding to the reactant and product states respectively. All parameters have units of kcal·mol^{-1}. Dihedral interactions (including improper dihedrals) were described by a periodic function of the form $E_{torsion} = V_1 \cdot (1 + \cos \varphi) + V_2 \cdot (1 - \cos 2\varphi) + V_3 \cdot (1 + \cos 3\varphi)$.

Table S6. Improper torsion types used to describe the two EVB states for the hydride transfer step of the oxidative deamination of histamine by MAO-B.[a]

**Atom number**				**State I**		**State II**	
**#1**	**#2**	**#3**	**#4**	k_a	τ_0	k_a	τ_0
7	5	4	6	10.5	180	1.1	180
16	7	5	8	10.5	180	1.1	180
23	20	21	22	*n.a.*	*n.a.*	1	180
7	8	9	24	*n.a.*	*n.a.*	1	180
26	23	25	20	*n.a.*	*n.a.*	10.5	180

[a] The units of k_a and τ_0 are given in kcal·mol^{-1}·rad^{-2} and °, respectively

Table S7. Nonbonding parameters used to describe the two EVB states during the direct hydride transfer in the oxidative deamination of N-methylhistamine by MAO-B.[a]

Atom number	State I			State II		
	A_i	B_i	q_i	A_i	B_i	q_i
1	885.43	27.02	-0.7443	971.75	28.31	-0.8619
2	1802.24	34.18	1.0090	1802.24	34.18	0.9920
3	616.44	23.77	-0.6117	616.44	23.77	-0.7546
4	971.75	28.31	-0.7738	971.75	28.31	-0.7680
5	1802.24	34.18	0.7294	1039.88	24.25	0.7563
6	616.44	23.77	-0.5553	976.93	31.26	-0.7333
7	1059.13	23.67	0.2576	1039.88	24.25	-0.4382
8	971.75	28.31	-0.5511	1064.97	29.63	-0.4260
9	1059.13	23.67	0.4846	1059.13	23.67	0.2654
10	1059.13	23.67	-0.3610	1059.13	23.67	-0.5285
11	1059.13	23.67	0.1155	1059.13	23.67	0.2023
12	1059.13	23.67	0.1463	1059.13	23.67	0.0387
13	1059.13	23.67	-0.2837	1059.13	23.67	-0.3640
14	1059.13	23.67	-0.1662	1059.13	23.67	0.0233
15	1064.97	29.63	0.0996	971.75	28.31	-0.0233
16	58.02	5.09	0.3831	1039.88	24.25	0.5280
17	0.00	0.00	0.4042	0.00	0.00	0.3553
18	69.58	4.91	0.2236	69.58	4.91	0.2061
19	69.58	4.91	0.1942	69.58	4.91	0.1982
20	1064.9718	29.6344	-1.1061	971.7502	28.3077	-0.5972
21	0.0054	0.0433	0.3896	0.0054	0.0433	0.4144
22	0.0054	0.0433	0.3896	0.0054	0.0433	0.4144
23	944.5180	22.0296	0.6832	1802.2385	34.1758	0.4365
24	59.8020	3.8273	-0.0796	0.00	0.00	0.3322
25	59.8020	3.8273	-0.0796	69.5797	4.9095	0.1311
26	944.5180	22.0296	-0.5259	944.5180	22.0296	-0.3185
27	84.5728	5.4127	0.1066	84.5728	5.4127	0.1543
28	84.5728	5.4127	0.1066	84.5728	5.4127	0.1543
29	1059.1297	23.6736	0.5480	1059.1297	23.6736	0.4010
30	1059.1297	23.6736	-0.5412	1059.1297	23.6736	-0.4700
31	69.5797	4.9095	0.2418	69.5797	4.9095	0.2713
32	971.7502	28.3077	0.1521	971.7502	28.3077	0.1924
33	944.5180	22.0296	-0.3352	944.5180	22.0296	-0.3248
34	84.5728	5.4127	0.1337	84.5728	5.4127	0.1507
35	84.5728	5.4127	0.1337	84.5728	5.4127	0.1507
36	84.5728	5.4127	0.1337	84.5728	5.4127	0.1507
37	1059.1297	23.6736	0.1384	1059.1297	23.6736	0.0907
38	69.5797	4.9095	0.1363	69.5797	4.9095	0.1848
39	971.7502	28.3077	-0.6321	971.7502	28.3077	-0.5889

[a] The atom numbering corresponding to valence bond states shown in Figure S2, with States I and II corresponding to the reactant and product states respectively. Van der Waals interactions were described by the 12-6 Lennard Jones potential, where A_i and B_i are single-atom (square-rooted) parameters in units of $kcal^{1/2}\cdot mol^{-1/2}\cdot Å^{-6}$ and $kcal^{1/2}\cdot mol^{-1/2}\cdot Å^{-3}$, respectively, while q_i is the partial atomic charge.

Table S8. Bonding parameters used to describe the two EVB states during the direct hydride transfer step of the oxidative deamination of N-methylhistamine by MAO-B.[a]

Atom number		State I		State II	
#1	#2	f_c	r_0	f_c	r_0
1	2	1000.00	1.261	914.00	1.358
1	16	1000.00	1.261	966.00	1.339
2	4	980.00	1.335	836.00	1.388
4	5	980.00	1.335	896.00	1.365
5	6	1140.00	1.229	900.00	1.370
5	7	938.00	1.400	1098.00	1.340
7	8	914.00	1.358	962.00	1.340
7	16	800.00	1.490	854.00	1.433
8	9	966.00	1.339	962.00	1.340
20	21	868.00	1.0100	980.00	1.0150
20	22	868.00	1.0100	980.00	1.0150
20	23	764.00	1.4480	1270.00	1.2860
23	25	680.00	1.0900	770.00	1.0830
23	26	536.00	1.5290	580.00	1.4880

		D_e	a	r_0	D_e	a	r_0
8	24	*n.a.*	*n.a.*	*n.a.*	100.60	2.000	1.100
23	24	102.76	2.000	1.100	*n.a.*	*n.a.*	*n.a.*

[a]The atom numbering corresponds to the atoms displayed in Figure S2, with States I and II corresponding to the reactant and intermediate states, respectively. Bonds were described with either a harmonic potential of the form $E_{bond} = ½\cdot f_c\,(r - r_0)^2$, or in the case of forming or breaking bonds, with a Morse potential of the form $E_{bond} = D_e\cdot(e^{-2a\cdot(r-r_0)} - 2e^{-a\cdot(r-r_0)})$. All f_c and D_e values are given in kcal·mol^{-1}, and r_0 values in Å.

Table S9. Angle parameters used to describe the two EVB states for the direct hydride transfer step of the oxidative deamination of N-methylhistamine by MAO-B.[a]

Atom number			State I		State II	
#1	#2	#3	f_c	θ	f_c	θ
1	2	3	160.00	120.600	160.00	122.500
1	2	4	140.00	114.200	140.00	118.600
1	16	15	140.00	124.100	140.00	119.300
2	1	16	100.00	120.500	140.00	120.500
2	4	5	140.00	126.400	140.00	121.600
2	4	17	70.00	119.800	70.00	116.800
3	2	4	160.00	122.900	160.00	120.600
4	5	6	160.00	122.900	160.00	120.600
4	5	7	140.00	116.600	140.00	121.200
5	4	17	70.00	119.800	70.00	119.200
5	7	8	160.00	123.000	140.00	121.200
5	7	16	160.00	117.200	170.00	117.000
6	5	7	160.00	121.400	140.00	123.00
7	8	9	140.00	120.500	100.00	109.500
7	8	24	*n.a.*	*n.a.*	70.00	111.00
8	7	16	160.00	123.00	140.00	120.100
8	9	10	140.00	124.00	140.00	120.100
8	9	14	140.00	124.00	140.00	120.100
9	8	24	*n.a.*	*n.a.*	70.00	111.00
20	23	24	70.00	109.500	*n.a.*	*n.a.*
20	23	25	70.00	109.500	90.00	118.0390
20	23	26	112.40	109.4700	140.00	120.6520
21	20	22	87.20	106.4000	60.00	117.8320
21	20	23	70.00	109.5000	90.00	118.8950
22	20	23	70.00	109.5000	90.00	118.8950
23	26	27	75.00	110.7000	90.00	109.3340
23	26	28	75.00	110.7000	90.00	109.3340
23	26	29	140.00	112.6240	140.00	112.3600
24	23	25	66.00	107.800	*n.a.*	*n.a.*
24	23	26	75.00	110.700	*n.a.*	*n.a.*
25	23	26	75.00	110.700	70.00	121.9150

[a] The atom numbering corresponding to the atom numbering used here are displayed in Figure S2, with States I and II corresponding to reactant and product states, respectively. The bending motion was described with a harmonic potential of the form $E_{angle} = f_c \cdot (\theta - \theta_0)^2$, where f_c is given in kcal·mol^{-1} and θ in °.

Table S10. Torsion parameters used to describe the two EVB states for the direct hydride transfer step of the oxidative deamination of N-methylhistamine by MAO-B.[a]

Atom number				State I			State II		
#1	#2	#3	#4	V_1	V_2	V_3	V_1	V_2	V_3
1	2	4	5	0.000	3.968	0.000	0.000	2.325	0.000
1	2	4	17	0.000	1.015	0.000	0.000	2.325	0.000
5	7	16	1	0.000	0.839	0.000	0.000	7.000	0.000
8	7	16	1	0.000	3.500	0.000	0.000	7.000	0.000
14	15	16	1	0.000	1.826	0.000	0.000	3.968	0.000
2	1	16	7	-1.183	0.456	-0.425	0.000	3.625	0.000
2	1	16	15	0.000	1.826	0.000	0.000	3.625	0.000
2	4	5	6	0.000	3.045	0.000	0.000	3.625	0.000
2	4	5	7	1.150	3.045	0.000	0.000	3.625	0.000
16	1	2	4	0.000	1.826	0.000	0.000	5.000	0.000
4	5	7	8	0.000	3.500	0.000	0.000	7.000	0.000
4	5	7	16	0.000	0.550	0.000	0.000	7.000	0.000
5	7	8	9	0.000	7.000	0.000	-3.791	1.715	1.599
5	7	8	24	*n.a.*	*n.a.*	*n.a.*	0.000	1.015	0.000
5	7	16	15	0.000	0.839	0.000	0.000	7.000	0.000
17	4	5	6	0.000	2.450	0.000	0.000	3.625	0.000
6	5	7	8	0.000	0.410	0.000	0.000	7.000	0.000
6	5	7	16	0.000	1.050	0.000	0.000	7.000	0.000
17	4	5	7	0.000	2.450	0.000	0.000	1.525	0.000
7	8	9	10	0.000	3.625	0.000	-3.791	1.715	1.599
7	8	9	14	0.000	3.625	0.000	-3.791	1.715	1.599
8	7	16	15	0.0000	3.500	0.000	0.000	7.000	0.000
16	7	8	9	0.000	7.000	0.000	-3.791	1.715	1.599
24	8	9	10	*n.a.*	*n.a.*	*n.a.*	0.000	1.015	0.000
24	8	9	14	*n.a.*	*n.a.*	*n.a.*	0.000	1.015	0.000
16	7	8	24	*n.a.*	*n.a.*	*n.a.*	0.000	1.015	0.000
20	23	26	27	0.1500	0	0	0	0	0
20	23	26	28	0.1500	0	0	0	0	0
20	23	26	29	0.8375	0.3220	0.7060	0	0	0
21	20	23	24	0.2000	0	0	*n.a.*	*n.a.*	*n.a.*
21	20	23	25	0.2000	0	0	2.000	0	0
21	20	23	26	-0.0595	0.0885	0.1175	2.000	0	0
22	20	23	24	0.2000	0	0	*n.a.*	*n.a.*	*n.a.*
22	20	23	25	0.2000	0	0	2.000	0	0
22	20	23	26	-0.0595	0.0885	0.1175	2.000	0	0
23	26	29	30	0.0415	0	0	0.1805	0	0
23	26	29	36	0.2930	-1.1970	0.6090	0.0430	0	0
24	23	26	27	0.1500	0	0	*n.a.*	*n.a.*	*n.a.*
24	23	26	28	0.1500	0	0	*n.a.*	*n.a.*	*n.a.*
24	23	26	29	0.4320	0	0	*n.a.*	*n.a.*	*n.a.*
25	23	26	27	0.1500	0	0	0.3310	0	0
25	23	26	28	0.1500	0	0	0.3310	0	0
25	23	26	29	0.4320	0	0	0	0	0

[a]The atom numbering corresponds to the atom numbers used in Figure S2, with States I and II corresponding to the reactant and product states respectively. All parameters have units of kcal·mol^{-1}. Dihedral interactions (including improper dihedrals) were described by a periodic function of the form $E_{torsion} = V_1 \cdot (1 + \cos \varphi) + V_2 \cdot (1 - \cos 2\varphi) + V_3 \cdot (1 + \cos 3\varphi)$.

Table S11. Improper torsion types used to describe the two EVB states for the direct hydride transfer step of the oxidative deamination of N-methylhistamine by MAO-B.[a]

**Atom number**				**State I**		**State II**	
**#1**	**#2**	**#3**	**#4**	$\boldsymbol{k_a}$	τ_0	$\boldsymbol{k_a}$	τ_0
7	5	4	6	10.5	180	1.1	180
16	7	5	8	10.5	180	1.1	180
23	20	21	22	*n.a.*	*n.a.*	1	180
7	8	9	24	*n.a.*	*n.a.*	1	180
26	23	25	20	*n.a.*	*n.a.*	10.5	180

[a]The units of k_a and τ_0 are given in kcal·mol^{-1}·rad^{-2} and °, respectively

Optimized transition state for reference reaction for direct hydride transfer from histamine to FAD of the MAO-B active site (Cartesian coordinates, in Å).

Transition state histamine (TS)

H	0.000881	0.080626	0.015165
N	-0.040777	0.006174	7.193443
C	0.408353	0.051443	8.492055
O	-0.305054	-0.135307	9.461829
N	1.770709	0.346668	8.702807
C	2.707618	0.617310	7.728365
O	3.852044	0.977934	7.995860
C	2.189026	0.520562	6.378068
N	3.067217	0.759291	5.359403
C	2.540167	0.639453	4.054232
C	3.397534	0.812414	2.967633
C	2.953145	0.721078	1.653680
C	3.905542	0.909665	0.501740
C	1.594083	0.429940	1.425339
C	1.069308	0.306420	0.018336
C	0.735174	0.248769	2.506457
C	1.182793	0.356075	3.831569
N	0.317497	0.177264	4.914791
C	0.801950	0.233145	6.209614
C	-1.073867	-0.176844	4.673003
H	-1.131978	-1.118769	4.117813
H	1.585002	-0.491218	-0.527483
H	1.222338	1.232323	-0.546817
H	3.965802	0.007383	-0.116742
H	4.911305	1.138256	0.860817
H	3.585483	1.727231	-0.153680
H	-0.306912	0.026970	2.306521
H	4.445587	1.011851	3.180587
H	2.070065	0.407282	9.668334
H	-1.560434	-0.291890	5.638073
H	-1.569954	0.611540	4.098177
N	1.953418	3.089941	6.559015
H	1.021763	3.077973	6.169386
H	2.022176	3.292936	7.552809
C	3.029991	3.287772	5.778529
H	2.823559	3.530187	4.735376
C	4.289497	3.815438	6.432774
H	5.093229	3.847195	5.693019
H	4.589652	3.117461	7.222216
C	4.067612	5.179140	7.022637
C	4.653438	6.378077	6.707312
H	5.403640	6.648388	5.980940

N	4.089661	7.294972	7.565491
H	4.302357	8.279250	7.612886
C	3.199683	6.627051	8.346269
H	2.616255	7.115493	9.113525
N	3.161134	5.351455	8.044984
H	3.482214	1.803506	5.490961

Optimized transition state for referece reaction for direct hydride transfer from N-methyhistamine to FAD of the MAO-B active site (Cartesian coordinates, in Å).

Transition state N-methylhistamine (TS)

H	-5.462051	-2.703650	-1.862245
N	-1.495574	3.166459	-0.681396
C	-0.577198	4.016760	-0.112655
O	-0.414701	5.171774	-0.464479
N	0.227229	3.508399	0.927763
C	0.195599	2.225856	1.433266
O	0.999540	1.828656	2.274224
C	-0.809940	1.381999	0.817581
N	-0.913372	0.101939	1.281001
C	-1.911693	-0.692161	0.675451
C	-2.095175	-1.997630	1.131175
C	-3.049331	-2.845459	0.581242
C	-3.222150	-4.250550	1.096536
C	-3.862744	-2.353605	-0.458155
C	-4.918767	-3.232565	-1.076304
C	-3.688952	-1.048668	-0.912283
C	-2.712272	-0.200976	-0.368665
N	-2.531827	1.104945	-0.833248
C	-1.592092	1.930973	-0.242152
C	-3.404025	1.625301	-1.876369
H	-4.446249	1.607057	-1.541267
H	-5.645380	-3.565946	-0.327399
H	-4.477037	-4.133255	-1.516816
H	-4.227265	-4.404908	1.504295
H	-2.502138	-4.466188	1.888875
H	-3.081961	-4.989827	0.300266
H	-4.324773	-0.691056	-1.713952
H	-1.466324	-2.335988	1.952041
H	0.913077	4.145619	1.313860
H	-3.104217	2.650077	-2.079777
H	-3.307428	1.022519	-2.784985
N	1.183723	0.520280	-0.605632

H	0.671968	0.594355	-1.472919
H	1.930064	1.195947	-0.468003
C	1.220882	-0.635715	0.081373
H	0.741315	-1.489296	-0.399582
C	2.391367	-0.869847	1.011568
H	2.221343	-1.788601	1.578734
H	2.439270	-0.040999	1.726420
C	3.681823	-0.958580	0.247622
C	4.556874	-2.009114	0.122363
H	4.551650	-3.009683	0.528420
N	5.577611	-1.553042	-0.679628
C	6.753724	-2.297928	-1.088122
C	5.274989	-0.268746	-1.002491
H	5.920999	0.331274	-1.629473
N	4.142378	0.122555	-0.464469
H	0.089465	-0.407232	1.140593
H	7.367914	-1.658914	-1.723589
H	7.338116	-2.595098	-0.214535
H	6.464245	-3.186465	-1.653553
